# An introduction to “Sugar and spice, and everything nice: exploring prosocial development through infancy and early childhood”

**DOI:** 10.3389/fpsyg.2014.01499

**Published:** 2014-12-23

**Authors:** Amanda Williams, Markus Paulus, Chris Moore

**Affiliations:** ^1^Department of Psychology and Neuroscience, Dalhousie UniversityHalifax, NS, Canada; ^2^Ludwig-Maximilians-Universität München, Department of PsychologyMunich, Germany

**Keywords:** prosocial behavior, development, sharing, helping, infancy, childhood

Prosocial behaviors begin to emerge at an early age (e.g., Damon, 1975; Rheingold et al., 1976; Eisenberg and Fabes, 1998; Thompson et al., 1997; Paulus, 2014). For example, helping behaviors are demonstrated by infants as young as 18 months of age (e.g., Warneken and Tomasello, 2006) and sharing behavior begins to emerge at around 2 years of age (e.g., Rheingold et al., 1976). Such prosociality is essential to social functioning in many respects. However, while prosocial behavior has long been of interest to developmental researchers, there remains much we do not know about the early development of prosocial behaviors.

This research topic builds on a well-established area of research, and brings together the work of various researchers in the field of prosocial development who have contributed unique theoretical perspectives, insightful reviews, and novel empirical work. The goal of this research topic is to examine broadly how, why, and when a spectrum of behaviors emerge, and enhance our understanding of the beginnings of human prosociality. Here, the existing literature is reviewed, and new insights into the development of prosocial behaviors are offered. A broad range of topics such as helping, cooperation, sharing, inequality aversion, and moral reasoning are covered, and various factors influencing prosociality explored.

As discussed in a theoretical contribution by Keith Jensen, Amrisha Vaish, and Marco Schmidt, prosociality is unique to humans and its development is influenced by a variety of mechanisms such as empathy, other-regarding concerns, and normativity. Importantly, the term prosocial behavior encompasses a multitude of behaviors, however, in her review article, Kristen Dunfield proposes that other-oriented, prosocial actions can be categorized into three specific subtypes; sharing, helping, and comforting, drawing on existing literature to support her proposal.

These subtypes of prosocial behaviors are further explored in this research topic using a variety of approaches. For instance, helping in early childhood is explored in a theoretical contribution by Stuart Hammond. While Hammond discusses the development of early helping behavior using a Piagetian framework, helping is also explored empirically by Sunae Kim, Beate Sodian, and Markus Paulus, who investigate differences in children's expectations regarding instrumental helping and self-helping at different points in development.

Other empirical contributions include work by Martina Vogelsang, Keith Jensen, Sebastian Kirschner, Claudio Tennie, and Michael Tomasello exploring cooperation in 5-year-olds using a public goods game, and a study by Sophia Ongley, Marta Nola, and Tina Malti demonstrating that moral reasoning is a strong predictor of the generosity of children's donations. Ongley and colleagues also explore how moral emotions such as sympathy and guilt relate to children's donating behavior, while several other experiments included in this research topic also explore various aspects of empathy and sympathy in early childhood. For instance, in a longitudinal study exploring the stability of sympathy over time, Jutta Kienbaum demonstrates that sympathy shows strong stability, and increases between the last year of preschool and the first year of school. Meanwhile, Jesse Drummond, Elena Paul, Whitney Waugh, Stuart Hammond and Celia Brownell show that empathic helping in toddlers is predicted by emotion and mental state talk, and Amanda Williams, Kelly O'Driscoll and Chris Moore demonstrate that experiencing empathic concern for another individual affects subsequent prosocial behavior; both decreasing envy, and increasing sharing.

Other factors influencing prosociality and social interactions are also investigated. In a study exploring the motivations underlying preferences for equality in situations of disadvantageous inequality, Amanda Williams and Chris Moore offer evidence that in some situations social comparison concerns—as opposed to fairness norms, influence decision-making. Markus Paulus demonstrates that the wealth of one's sharing partner also influences 5-year-olds sharing behavior with that individual, while Monica Burns and Jessica Sommerville demonstrate that both fairness and race influence 15-month-old infants' selection of social partners. In a study looking at social preferences in an even younger sample, Kiley Hamlin demonstrates that even infants as young as 4.5 months display context-dependent social preferences, and selectivity in prosocial behavior is further discussed in a review paper by Valerie Kuhlmeier, Kristen Dunfield, and Amy O'Neill.

Together this body of research demonstrates and discusses the complexities of a wide range of prosocial behaviors. It makes a significant contribution to the extant literature across a range of age groups, and a wide breadth of topics. It is our hope that the novel ideas, methodologies, and findings presented here help us better understand the development of early prosocial behavior as a whole, and stimulate and inspire future research.

## Conflict of interest statement

The authors declare that the research was conducted in the absence of any commercial or financial relationships that could be construed as a potential conflict of interest.
